# Comparison of 2D and 3D radiomics features with conventional features based on contrast-enhanced CT images for preoperative prediction the risk of thymic epithelial tumors

**DOI:** 10.2478/raon-2025-0016

**Published:** 2025-02-27

**Authors:** Yu-Hang Yuan, Hui Zhang, Wei-Ling Xu, Dong Dong, Pei-Hong Gao, Cai-Juan Zhang, Yan Guo, Ling-Ling Tong, Fang-Chao Gong

**Affiliations:** 1 Department of Radiology, The First Hospital of Jilin University, Jilin, China; 2 GE Healthcare, China; 3 Department of Pathology, The First Hospital of Jilin University, Jilin, China; 4 Department of Thoracic Surgery, The First Hospital of Jilin University, Jilin, China

**Keywords:** thymic epithelial tumors, radiomics, computed tomography, World Health Organization, classification

## Abstract

**Background:**

This study aimed to develop and validate 2-Dimensional (2D) and 3-Dimensional (3D) radiomics signatures based on contrast-enhanced computed tomography (CECT) images for preoperative prediction of the thymic epithelial tumors (TETs) risk and compare the predictive performance with conventional CT features.

**Patients and methods:**

149 TET patients were retrospectively enrolled from January 2016 to December 2018, and divided into high-risk group (B2/B3/TCs, n = 103) and low-risk group (A/AB/B1, n = 46). All patients were randomly assigned into the training (n = 104) and testing (n = 45) set. 14 conventional CT features were collected, and 396 radiomic features were extracted from 2D and 3D CECT images, respectively. Three models including conventional, 2D radiomics and 3D radiomics model were established using multivariate logistic regression analysis. The discriminative performances of the models were demonstrated by receiver operating characteristic (ROC) curves.

**Results:**

In the conventional model, area under the curves (AUCs) in the training and validation sets were 0.863 and 0.853, sensitivity was 78% and 55%, and specificity was 88% and 100%, respectively. The 2D model yielded AUCs of 0.854 and 0.834, sensitivity of 86% and 77%, and specificity of 72% and 86% in the training and validation sets. The 3D model revealed AUC of 0.902 and 0.906, sensitivity of 75% and 68%, and specificity of 94% and 100% in the training and validation sets.

**Conclusions:**

Radiomics signatures based on 3D images could distinguish high-risk from low-risk TETs and provide complementary diagnostic information.

## Introduction

Thymic epithelial tumors (TETs) are the most prevalent neoplasms in the anterior mediastinum. They generally exhibit low malignancy in both histological and clinical presentations.^1^ The WHO classification of TETs, based on lymphocyte-to-epithelial cell ratio and epithelial cell morphology, is widely adopted. According to the 2015 criteria, TETs are categorized into thymomas (with six subtypes) and thymic carcinomas (TCs).^2,3^ This classification serves as an independent prognostic factor and is simplified into low-risk (Type A, AB, B1) and high-risk (Type B2, B3, TCs) categories, influencing patient outcomes.^4^ The WHO classification reflects the tumor’s clinical and functional features, aiding preoperative diagnosis and treatment planning.^5^ Accurate, noninvasive identification and subgroup classification of TETs prior to treatment hold significant clinical value.

CT imaging is the primary diagnostic tool for TETs, revealing a wide range of biological and morphological features.^6,7^ Studies have reported specific CT characteristics of TETs.^8,9^ While chest contrast-enhanced computed tomography (CECT) provides general morphological parameters, distin-guishing between histological subgroups remains challenging due to significant overlap. Radiomics, leveraging radiomic signatures, extracts diverse, high-throughput imaging features, transforming medical images into mineable data.^10,11^ Radiomic features can predict disease, cancer, metastasis, and prognosis.^12^ Previous studies have used 2D radiomics to determine thymoma risk levels.^13,14^ Wang *et al*. differentiated low-risk from high-risk thymomas and early from advanced tumors using contrast-enhanced CECT and non-enhanced CT15, but their study had a small sample size and the sample size was imbalanced.

Despite several CT-based radiomics analyses being used to classify TET risk, most studies rely on 2D imaging features. Further research is needed to explore 3D radiomic signatures, which may provide more comprehensive and accurate information for TET diagnosis and risk assessment. The available models still require refinement and validation.

This study aimed to develop conventional and 2D/3D radiomics signatures using the conventional and texture features extracted from CECT images for preoperative prediction of the WHO’s TET risk classification.

## Patients and methods

### Patients

**FIGURE 1.**
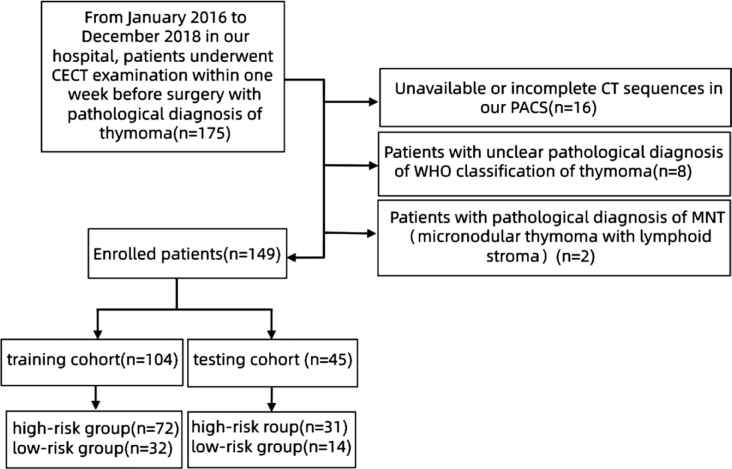
The flow chart of the case selection process

This retrospective study was approved by the Ethics Committee of the First Hospital of Jilin University, and the requirement for informed consent was waived due to the retrospective nature of the study (Studi Approval Number: 2020-541). From January 2016 to December 2018, data from 175 patients who undergoing CECT examination within one week to two months before surgery who were pathologically diagnosed as TET were collected in our hospital consecutively. The inclusion criteria were: 1) underwent CECT within one week to two months before surgery without chemotherapy; 2) high CT image quality without artifacts; and 3) available clinical and surgical data. The exclusion criteria were shown in Figure 1. Finally, 149 patients with pathologically confirmed TET were enrolled in this study. The baseline characteristics of all patients including age, sex and symptoms (thoracalgia and myasthenia gravis) were collected. The patients were divided into the high-risk group (B2/B3/TCs, n = 103) and the low-risk group (A/AB/B1, n = 46) according to the WHO classification criteria of TET. All patients were randomly assigned into the training group (n = 104) and test (n = 45) group at a ratio of 7:3. Figure 1 illustrates the flow chart of the case selection process.

### CT scanning

All patients underwent routine two-phase chest CECT scans using either a 64-MDCT scanner (Definition, Siemens Healthcare, Erlangen, Germany) or a 128-MDCT scanner (iCT, Philips Healthcare, Amsterdam, Netherlands). The scanning parameters were as follows: (1) Philips iCT: tube voltage 120 kV, automatic mAs, layer thickness 5 mm, pitch 0.980; (2) GE Lightspeed VCT: tube voltage 120 kV, automatic mAs, slice thickness 5 mm, pitch 0.992. All patients were examined in a supine position, arms up, deep inspiration and scanning. The contrast agent was injected into the patient using a high-pressure syringe (Visipaque 320, Amersham Health, Cork, Ireland). A total of 60-80 mL of contrast agent was administered through the antecubital vein at a rate of 3 ml/s, which was followed by 30 ml of saline injection at the same rate.

### Conventional CT features measurement

**Figure 2.**
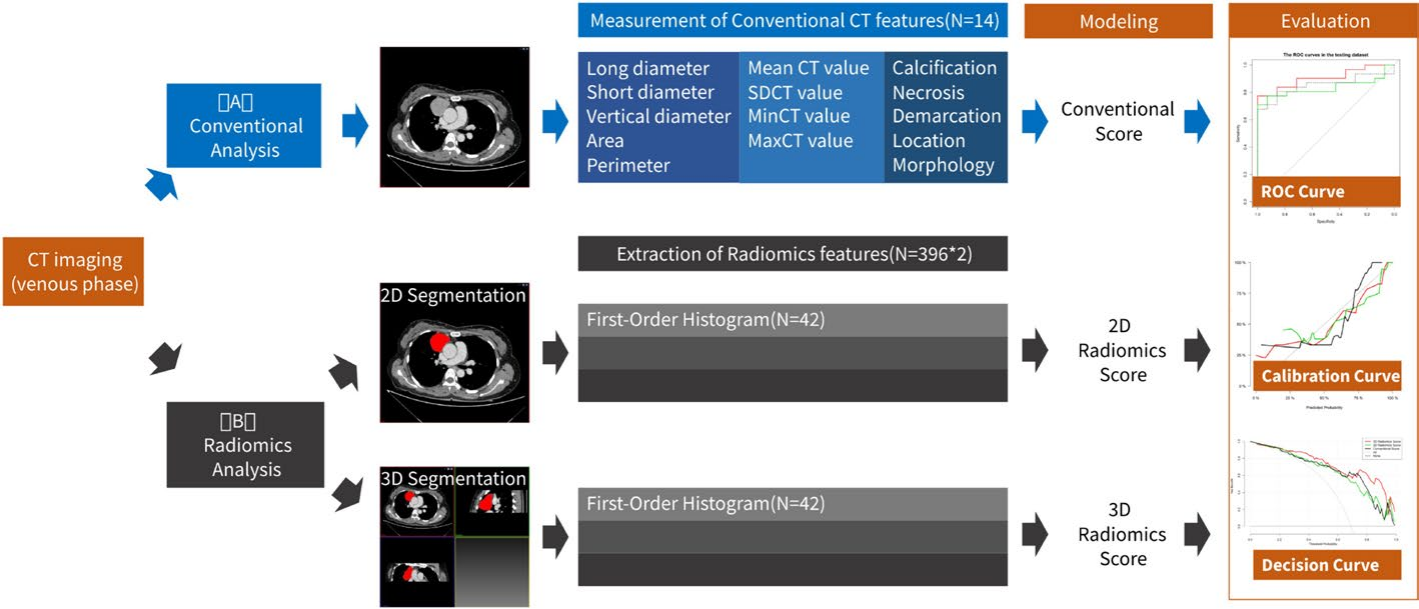
The flow chart of the CT imaging analysis. **(A)** shows the workflow of conventional analysis and 14 conventional features were recorded. **(B)** shows the workflow of radiomics analysis. 2D and 3D segmentation were performed on the CT images and 396 radiomics features were extracted respectively. The most predictive feature variables were selected, and the multivariate logistic regression analysis was applied to build the prediction models. The predicting abilities of the conventional and radiomics models were demonstrated by receiver operating characteristic (ROC) curves. The goodness of fit was assessed using calibration curve of the Hosmer-Lemeshow test. Additionally, decision curve analysis (DCA) was conducted to determine the clinical usefulness of the models.

The CT images were reviewed on the picture archiving and communication system (PACS). The conventional CT features of all patients were analyzed and recorded, including tumor’s long diameter, short diameter, vertical diameter, area, perimeter and CT values. The tumor heterogeneity evaluated by the radiologist is also recorded, consisting of location (right, middle or left), morphology (lobular,/shallowly-lobulated or non-lobular), demarcation (clear, unclear or infiltrating), internal calcification and necrosis. The workflow of conventional CT feature analysis was shown in Figure 2.

### 2D and 3D Tumor segmentation

The original CT images with a thickness of 5 mm were uploaded to the A.K. software (Artificial Intelligence Kit, A.K., GE Healthcare, China) for 2D and 3D manual segmentation. First, using a linear interpolation algorithm, the raw data were resampled to a common voxel spacing of 1 mm3 to construct new data points within the range of a discrete set of known data points, and the voxel would be isotropic. The data with a window width of 350 HU and a window level of 50 HU were used. The 2D and 3D regions of interest (ROIs) were delineated by two independent experienced radiologists (reader 1, XWL with 15 years of experience in chest imaging and reader 2, ZH with 10 years of experience in chest imaging) who were blinded to the pathology results. The 2D ROIs were delineated at the level of the single largest cross-sectional area around the tumor outline. The 3D ROIs were achieved from different continuous levels. To segment the ROI in axial CT images, a manual method was used on the AK software. The lesion was manually separated from the large blood vessels, lung, air, fat tissue and chest wall. The workflow of radiomics analysis was shown in Figure 2.

### 2D and 3D radiomic features extraction

According to the CECT images of each patient, a total of 396 radiomic features were extracted automatically from the 3D and 2D ROI respectively using A.K. software. The radiomic features were composed of the following classes: first-order histogram features (N = 42), second-order texture features (N = 345, including the Haralick texture (N = 10), gray level co-occurrence matrix (N = 144), gray level run length matrix (N = 180) and gray level size zone matrix (N = 11), and morphological features (N = 9). Details of the radiomics features were described in Supplementary Figure. S1.

### Features selection

To reduce the dimensionality of the conventional and 2D/3D radiomic features, the least absolute shrinkage and selection operator (LASSO) method was applied to identify the most valuable features from the training dataset. For better performance of the model, the best penalty parameters λ was obtained based on the loss function with the least squares during 10-fold-cross-validation procedure.

### Model building and evaluation

Three models, including the conventional model, 2D radiomics model and 3D radiomics model, were established using multi-variate logistic regression based on the selected features. Then the conventional score, 2D radiomics score and 3D radiomics score were calculated for each patient via a linear combination of selected features weighted by coefficients.

The discriminative performance of the three models were evaluated by ROC curves. The area under the curves (AUC), sensitivity, specificity and the optimal cutoff value were obtained from ROC analysis. Delong test was used to identify the difference of AUC between different models, and P < 0.05 indicated a significant difference. The degree of calibration was assessed in the calibration curve of the Hosmer-Lemeshow test, and P > 0.05 indicated a good fit. Furthermore, decision curve analysis (DCA) was conducted to determine the clinical applicability of the models by quantifying the net benefit under different threshold probabilities.

### Statistical analysis

Continuous data with a normal distribution (according to the Kolmogorov-Smirnov test) were expressed as mean ± standard deviation (SD) and analyzed using Student’s t-test. Continuous variables with a non-normal distribution were expressed as medians (interquartile range) and analyzed using the Mann-Whitney test. Categorical variables were represented as frequencies and percentages and analyzed using the chi-square test. The statistical analysis was conducted using R software (v. 3.6.1, http://www.R-project.org). A two-tailed p value of less than 0.05 was considered significant.

## Results

### Baseline characteristics of the patients

There were 104 patients in the training set, among which 72 (69.2%) cases were in the high-risk group. The testing set involved 45 patients, and 31 (68.9%) cases were in the high-risk group. Age, sex, and frequency of myasthenia gravis and thoracalgia were similar between the high-risk and low-risk groups in both the training and testing sets (all P > 0.05). The characteristics of the patients are presented in Table 1.

**Table 1.** Baseline characteristics of the patients in training and testing dataset

	Training set			Testing set		
	Low-risk (n=32)	High-risk (n= 72)	P	Low-risk (n=14)	High-risk (n=31)	P
Age, (Mean ± SD) years	53.6±11.2	52.5±11.4	0.656	54.0±10.7	56.4±8.9	0.446
Sex (male, No. (%))	14 (43.8)	37 (51.4)	0.472	7 (50.0)	18 (58.1)	0.614
Myasthenia gravis, No. (%)	7 (21.9)	24 (33.3)	0.238	0 (0.0)	8 (25.8)	0.094
Thoracalgia, No. (%)	3 (9.4)	18 (25.0)	0.067	1 (7.1)	11 (35.5)	0.104

### Three models building

#### Conventional model

In the training set, compared with the low-risk group, the high-risk group had a lower mean HU value (62.0 *vs*. 79.5 HU, P < 0.001), a lower maximum HU value (118.0 *vs*. 148.5 HU, P < 0.001), a smaller short diameter 23.2 *vs*. 34.7 mm, P = 0.009), a smaller area (628.5 *vs*. 1321.5 mm^2^, P = 0.008), and a smaller perimeter (112.5 *vs*. 143.0 mm, P = 0.021). In addition, the lesions in the high-risk group were often non-lobular (P = 0.010), and there was no clear demarcation (P = 0.023). The patient’s CT conventional parameters are listed in Table 2.

**Table 2.** Distribution of conventional CT features in training and testing dataset

	Testing set			Training set		
	Low-risk (n=32)	High-risk (n= 72)	P	Low-risk (n=14)	High-risk (n=31)	P
Mean CT value (HU)	79.5 (68.4, 91.6)	62.0 (51.0, 78.8)	**<0.001**	88.5 (76.0, 95.1)	67.0 (59.2, 74.0)	**<0.001**
Standard deviation	18.0 (16.0, 22.6)	16.5 (14.0, 19.0)	0.050	18.0 (15.9, 26.2)	17.0 (14.2, 21.6)	0.548
Minimum CT value (HU)	-6.10±30.2	-8.5±25.0	0.678	-0.7±31.6	-7.3±17.9	0.477
Maximum CT value (HU)	148.5 (131.0, 172.1)	118.0 (105.5, 138.6)	**<0.001**	162.0 (148.9, 166.1)	129.0 (105.4, 146.8)	**0.002**
Long diameter (mm)	50.6±17.0	44.1±19.4	0.106	47.8 (40.9, 57.8)	38.0 (27.7, 61.3)	0.198
Short diameter (mm)	34.7 (23.9, 41.7)	23.2 (17.7, 34.6)	**0.009**	36.5 (26.0, 45.4)	25.6 (19.0, 39.3)	0.073
Vertical diameter (mm)	48.6 (44.1, 60.2)	40.4 (29.1, 55.2)	0.204	50.5 (44.4, 63.6)	38.9 (33.1, 55.1)	0.059
Area (mm^2^)	1321.5 (692.0, 1889.8)	628.5 (397.4, 1409.7)	**0.008**	1024.0 (747.7, 1623.3)	651.0 (346.0, 1362.8)	0.315
Perimeter (mm)	143.0 (110.8, 167.7)	112.5 (78.6, 153.8)	**0.021**	143.5 (118.6, 255.4)	100.0 (84.1, 194.8)	0.098
Location			0.373			0.790
Right mediastinum	10 (31.3%)	33 (45.8%)		7 (50.0%)	11 (35.5%)	
Middle	8 (25.0%)	15 (20.8%)		1 (7.1%)	3 (9.7%)	
Left mediastinum	14 (43.8%)	24 (33.3%)		6 (42.9%)	17 (54.8%)	
Morphology			**0.010**			**<0.001**
Lobular	5 (15.6%)	10 (13.9%)		7 (50.0%)	2 (6.5%)	
Shallowly-lobulated	15 (46.9%)	14 (19.4%)		7 (50.0%)	15 (48.4%)	
Non-lobular	12 (37.5%)	48 (66.7%)		0 (0.0%)	14 (45.2%)	
Demarcation			**0.023**			**0.010**
Clear	15 (46.9%)	17 (23.6%)		10 (71.4%)	8 (25.8%)	
Unclear	16 (50.0%)	43 (59.7%)		4 (28.6%)	17 (54.8%)	
Infiltration	1 (3.1%)	12 (16.7%)		0 (0%)	6 (19.4%)	
Internal calcification	8 (25.0%)	13 (18.1%)	0.416	4 (28.6%)	9 (29.0%)	0.746
Necrosis	12 (37.5%)	20 (27.8%)	0.321	9 (64.3%)	12 (38.7%)	0.111

1 Continuous variables that conformed to the normal distribution were expressed as mean ±SD. Continuous variables that did not conform to the normal distribution were represented by median values (25%, 75%). Categorical variables were expressed as No. (%).

1 Coarse P values represented statistically significant.

In the testing set, the maximum HU value of the high-risk group was lower than that of the low-risk group (median HU 129.0 *vs*. 162.0, P = 0.002). The lesions in the high-risk group tended to be non-lobular (P < 0.001), with no clear demarcation(P = 0.010).

After LASSO regression analysis, seven conventional CT features with non-zero coefficients were remained. The calculation formula of the conventional score was as follows:

Conventional Score = 5.71-0.012*Circle.mm-0.051*Mean CT value + 0.023*Minimum CT value-0.009*Maximum CT value-0.903*Necrosis + 1.859*adjacent-0.595*Location.

### 2D Radiomics model

After LASSO regression analysis, five CT features including Min Intensity, Percentila75, Correlation_ angle0_offset7, LongRunEmphasis_angle90_off-set1, and Small Area Emphasis were retained. The formula for the calculation of the 2D radiomics score was as follows:

2D Radiomics Score = 1.343-0.528 *Min intensity-0.805* Percentile75-0.557* Correlation_angle0_ offset7 + 1.343* LongRunEmphasis_angle90_offset 1-0.900* SmallAreaEmphasis

### 3D radiomics model

The 3D radiomics model was established using eight selected CT features, and the calculation formula of the 3D radiomics score was as follows:

3D Radiomics Score = 1.330-1.731 *Percentile75-0.915* ClusterProminence_angle135_offset4-1.266 *GLCMEnergy_angle0_off-set7 + 0.635* AngularSecondMoment-0.559 *LongRunEmphasis_AllDirection_offset1 + 0.678* Low GreyLevel RunEmphasis_AllDirection_ offset7_SD-0.573 *Compactness1-0.714* IntensityVariability.

### The performance of the three models

#### Discriminative degree

**Figure 3.**
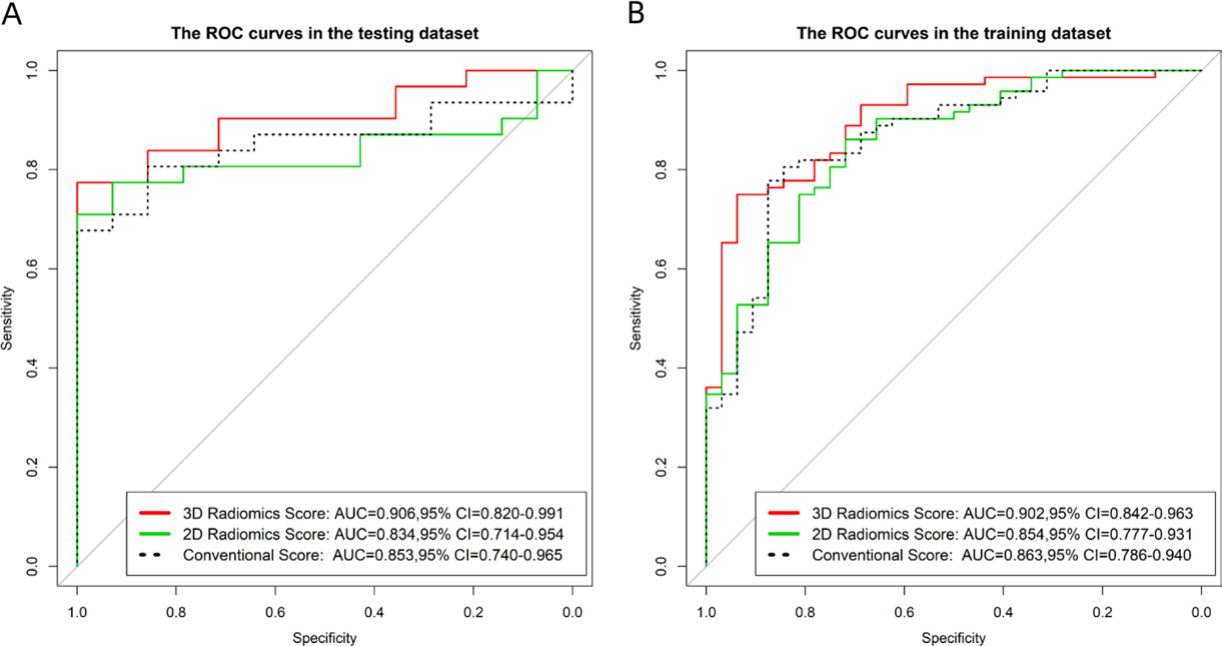
Receiver operating characteristic (ROC) curve analysis of the conventional, 2D and 3D radiomics models: **(A)** the training set; **(B)** the testing set.

In the training set, this conventional model had an AUC of 0.863 (95% confidence interval (CI): 0.786-0.940), a sensitivity of 78%, and a specificity of 88%. In the testing set, the AUC was 0.853 (95% CI: 0.740-0.965), the sensitivity was 55%, and the specificity was 100%. The 2D radiomics model yielded an AUCs of 0.854 (95% CI: 0.7-0.931) and 0.834 (95%CI: 0.714-0.954), a sensitivity of 86% and 77%, and a specificity of 72% and 86% in the training and testing set, respectively. For the 3D radiomics model, the AUC of the training and testing set was 0.902 (95%CI: 0.842-0.963) and 0.906 (95%CI: 0.820-0.991), respectively, the sensitivity was 75% and 68%, respectively, and the specificity was 94% and 100%, respectively. The ROC curves and the detailed results were shown in Figure 3 and Table 3.

**Table 3.** Diagnostic performance of the three models

Model	Training dataset			Testing dataset		
	Sensitivity	Specificity	AUC (95%CI)	Sensitivity	Specificity	AUC (95%CI)
Conventional models	77.8%	87.5%	0.863(0.786-0.940)	54.8%	100.0%	0.853(0.740-0.965)
2D radiomics model	86.1%	71.9%	0.854(0.777-0.931)	77.4%	85.7%	0.834(0.714-0.984)
3D radiomics model	75.0%	93.8%	0.902(0.842-0.963)	67.7%	100.0%	0.906(0.820-0.991)

1 AUC = area under the curve; CI = confidence interval; CT = computed tomography

As shown in Figure 3, the AUC of 3D model was usually higher compared with the conventional parameter model and 2D model. However, due to the limited data size, Delong test suggested that there was no statistical difference of AUCs among conventional, 2D and 3D models. The process and results of the Delong test are described in Supplementary Figure S2.

#### Calibration degree

**Figure 4.**
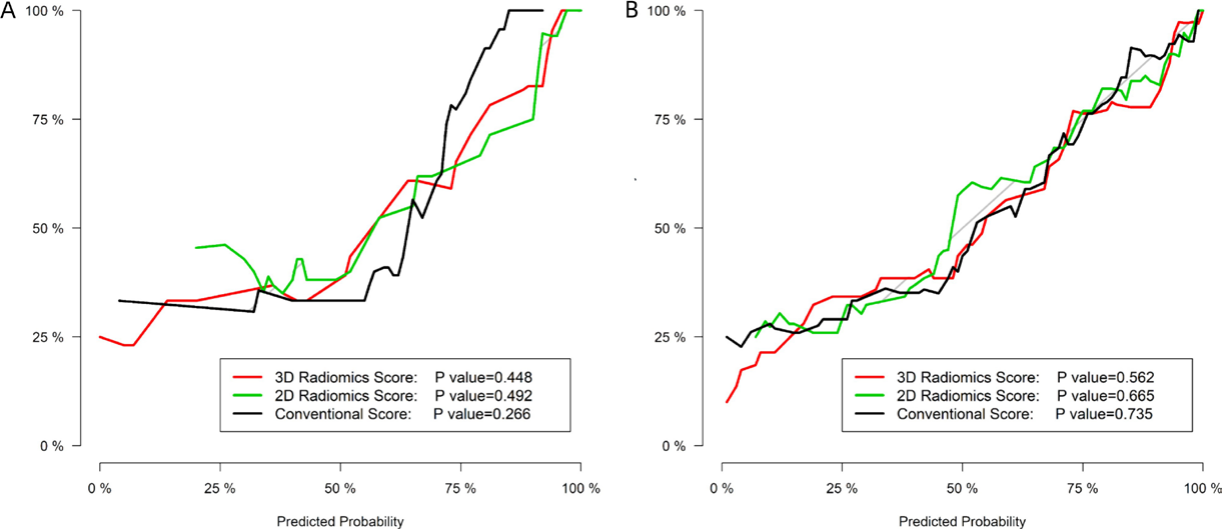
The calibration curves of the conventional, 2D and 3D radiomics models:**(A)** the training set; **(B)** the testing set.

The calibration curves of the three models were illustrated in Figure 4, which demonstrated good agreement between the predictive and actual probability. All the P value of the Hosmer-Lemeshow test were more than 0.05 in both training and testing datasets for the conventional model (P = 0.735 and 0.266, respectively), 2D model (P = 0.665 and 0.492, respectively) and 3D model (P = 0.562 and 0.448, respectively).

#### Clinical usefulness

**Figure 5.**
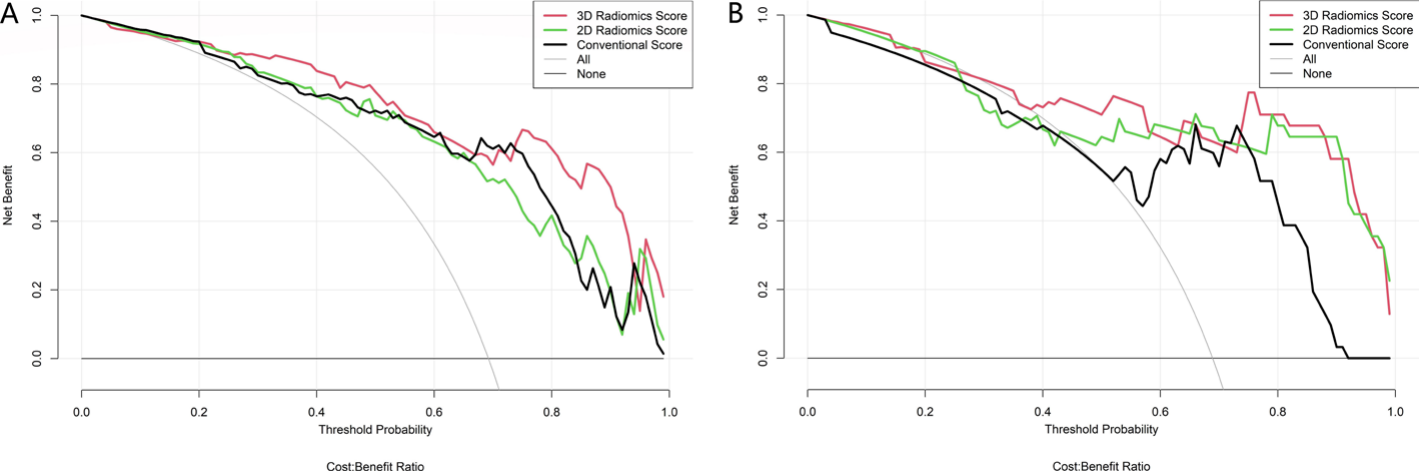
Decision curve analysis (DCA) to determine the clinical usefulness of the models by quantifying the net benefits under different threshold probabilities: **(A)** the training set; **(B)** the testing set.

DCA for the three models was performed in both training and testing datasets (Figure 5). The decision curve showed that all the three models added more benefit than using treat-all scheme (assuming all TETs are high risk) or the treat-none scheme (assuming all TETs are low risk) if the threshold probability was > 10%. When the threshold probability was betwen 25% to 60% or between 70% to 95%, using the 3D radiomics model to predict the TETs risk added more benefit than either the con-ventional model or the 2D radiomics model in both training and testing datasets.

## Discussion

This study evaluates three models using conventional and radiomic signatures, revealing that radiomic features from 2D and 3D imaging serve as noninvasive biomarkers for thymic epithelial tumors (TETs). These features enable preoperative risk stratification, guiding the surgical approach and resection extent. Importantly, risk stratification aids in personalized treatment planning for high-risk patients, often needing adjuvant therapy. A Phase II studies show that preoperative chemotherapy and radiotherapy enhance R0 resection rates.^16^ Thus, this study highlights the significance of radiomic signatures in preoperative risk assessment and treatment planning for TETs.

In this study, three established models obtained using the conventional and radiomics signatures were examined. The results indicated that the radiomics signatures based on conventional, 2D and 3D images could be used as noninvasive biomarkers to differentiate high-risk from low-risk TETs. Specifically, these models can provide preoperative risk stratification which informs the choice of surgical approach (minimally invasive surgery or thoracotomy) and helps define the extent of resection. By accurately predicting the risk, this model could aid in minimizing unnecessary tissue damage and reducing complications, potentially improving surgical outcomes and postoperative recovery. Since high-risk TET patients generally have poorer prognosis and often require adjuvant therapy, this stratification can guide treatment planning and improve patient outcomes by facilitating more personalized interventions.

Radiomics has shown significant potential in personalized medicine by providing non-invasive insights into tumor characteristics.^10,12^ It has been applied in various diseases, such as predicting thyroid cancer nodules, differentiating the benign and malignant nature of pulmonary nodules, and supporting clinical decision-making for liver cancer patients. Radiomics studies using different imaging modalities (including ultrasound, MRI, and CT) have increasingly played a crucial role in clinical decision-making, providing new technological support for prediction, diagnosis, and prognosis.^17,18,19^

Radiomics can be used to determine the TET classification according to WHO criteria. Previous studies have applied radiomics to classify the risk levels of thymomas and predict their invasiveness^15,20,21^, but the available models still need refinement and validation. Therefore, this study aimed to use radiomics based on CECT images to develop conventional and radiomics signatures, which may help the preoperative prediction of the risk of TET classified by WHO guideline.

We used a simplified classification that defines Type A, AB, and B1 thymomas as low-risk and Type B2 and B3 thymomas and TC as high-risk, which is related to patient outcome. High-risk thymic epithelial tumors reportedly have a much poorer prognosis compared with low-risk thymic epithelial tumors, the former need adjuvant therapy to improve survival. With complete resection of the tumor, the prevalence of recurrence of LTET seems low.

Our 3D radiomics model performed better in terms of AUC, sensitivity, and specificity compared to prior models reported in the literature. For instance, Wang *et al*. reported an AUC of 0.801, with a sensitivity of 75% and specificity of 77%, while Sui *et al*. found sensitivities ranging from 71% to 74% and specificities from 65% to 74% for differentiating low-risk and high-risk TETs. The improved performance of our model may be attributed to the enhanced information captured by 3D imaging features, which provide a more comprehensive representation of tumor morphology and texture heterogeneity than 2D features. This suggests that using 3D data could offer a substantial advantage in capturing the complex structural characteristics of TETs, which is consistent with findings from previous studies on the added value of 3D features. However, it should also be noted that 3D feature extraction and analysis increase computational complexity, highlighting a potential trade-off between accuracy and practicality. In this study, the images with 5 mm thickness in CECT were used for radiomics analysis, which not only saves time but also yields satisfactory results.

Due to the small sample size, the fluctuation of individual samples affected the results greatly. The Hosemer-Lemeshow test was greater than 0.05. Although there were small fluctuations in the calibration map images of the test set, it still showed good consistency between predicted probability and true possibility without statistical deviation.

In the clinical usefulness, the 3D radiomics model was valid from 25% to 60% and from 70% to 95% for the training set, and valid from 25% to 57% and from 75% to 90% for the test set. The DCA analysis showed the 3D model to be more effective and reliable than the other two models to determine the clinical management.

Conventional features such as contour, capsule, septum and homogeneous enhancement can be used to distinguish low-risk and high-risk TETs.^7–9^ Our study also compared the parameters obtained through the conventional CT features rather than software calculations, which also has some clinical significance for distinguishing high-risk from low-risk groups.

Except CT, magnetic resonance imaging (MRI) could also be used to differentiate the grading of TETs according to WHO criteria.^22,23^ Nevertheless, the use and combination of multiple imaging modalities could maximize the diagnostic value, provided that all modalities are available at a given hospital, but this is not always the case, especially in developing countries like China.

This study has several limitations. First, it is a single-center, small-sample, retrospective study, which may limit the generalizability of the findings and lead to overfitting. The small sample size also means that individual sample fluctuations could have a more significant impact on the model’s performance, thus affecting its robustness. To address this, we plan to expand the sample size and conduct a multi-center study for further investigation. Second, the absence of Masaoka staging information limits the model’s applicability in distinguishing between different stages of thymic epithelial tumors (TETs), which is crucial for clinical decision-making. In future studies, we will incorporate this information. Third, variability in CT scan parameters and reconstruction techniques could affect the consistency and reproducibility of radiomics features, further impacting model performance. Standardizing imaging acquisition and processing methods will be essential in future studies to enhance both model robustness and clinical applicability. Additionally, AI-based automated recognition methods will be employed for more in-depth tumor analysis. Although the model demonstrates excellent performance in distinguishing risk groups, additional factors, such as tumor heterogeneity or genetic information, may further enhance predictive accuracy^24^.

## Conclusions

In conclusion, radiomics signatures based on conventional, 2D and 3D images could be used as noninvasive biomarkers to differentiate high-risk TETs from low-risk TETs. The 3D radiomics signature can provide complementary diagnostic information and as the most useful method to determine the clinical management.

## Supplementary Material

Supplementary Material Details
