# Supplementary material for: Comparison of 2D and 3D radiomics features with conventional features based on contrast-enhanced CT images for preoperative prediction the risk of thymic epithelial tumors
Source: Radiol Oncol. 2025 Feb 27;59(1):69–78. doi: 10.2478/raon-2025-0016 (PMC11867572; doi:10.2478/raon-2025-0016)
Supplement: Supplementary file 1 — Supplementary Material Details [file raon-2025-0016_sm.pdf]

Comparison of 2D and 3D radiomics features with conventional features based on contrast-enhanced CT images for preoperative prediction the risk of thymic epithelial tumors

Yu-Hang Yuan, Hui Zhang, Wei-Ling Xu, Dong Dong, Pei-Hong Gao, Cai-Juan Zhang, Yan Guo, Ling-Ling Tong, Fang-Chao Gong

doi: 10.2478/raon-2025-0016

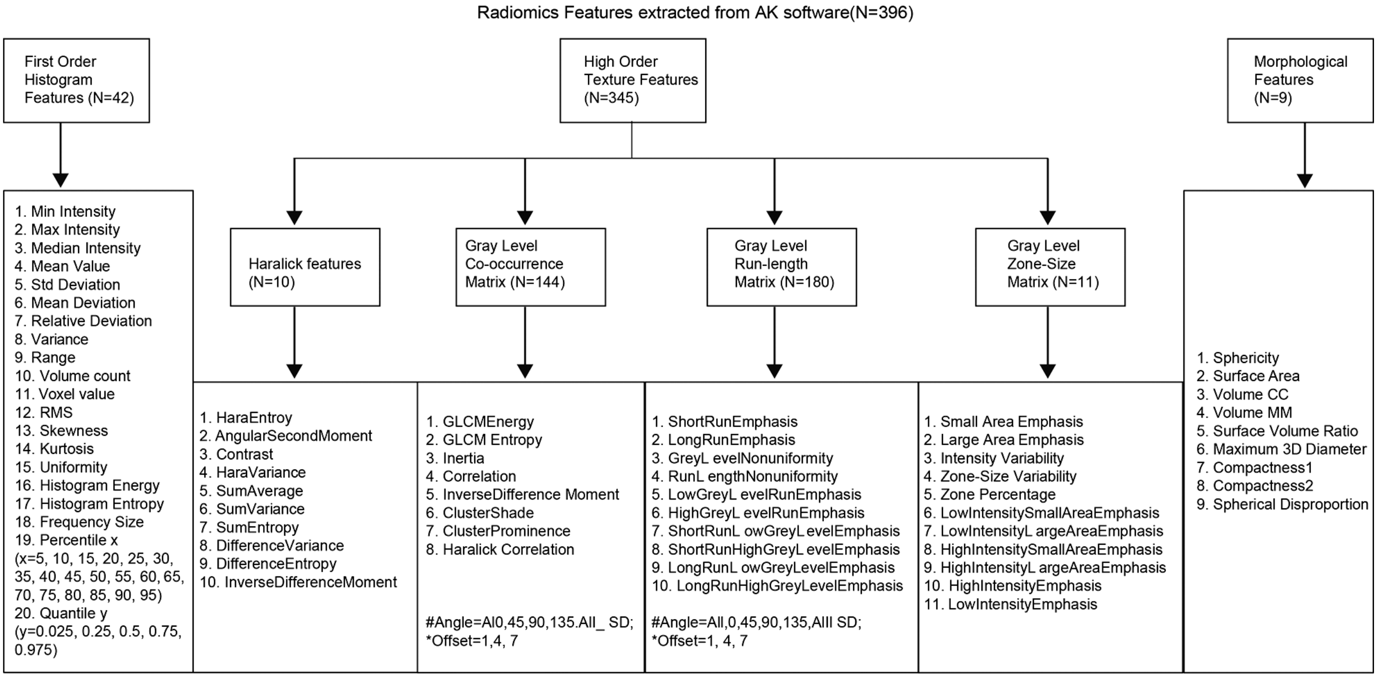

SUPPLEMENTARY FIGURE S1. radiomics features extracted from AK software

```
> roc.test(roc_train_2d,roc_train_3d)$p.value
[1] 0.237
> roc.test(roc_train_2d,roc_train_conv)$p.value
[1] 0.822
> roc.test(roc_train_3d,roc_train_conv)$p.value
[1] 0.326
> roc.test(roc_test_2d,roc_test_3d)$p.value
[1] 0.0909
> roc.test(roc_test_2d,roc_test_conv)$p.value
[1] 0.794
> roc.test(roc_test_3d,roc_test_conv)$p.value
[1] 0.395
> roc.test(roc_train_2d,roc_test_2d)$p.value
[1] 0.788
> roc.test(roc_train_3d,roc_test_3d)$p.value
[1] 0.953
> roc.test(roc_train_conv,roc_test_conv)$p.value
[1] 0.882
```

SUPPLEMENTARY FIGURE S2. The process and results of the Delong test among conventional, 2D and 3D models
